# Recent Progress of the ARegPKD Registry Study on Autosomal Recessive Polycystic Kidney Disease

**DOI:** 10.3389/fped.2017.00018

**Published:** 2017-02-16

**Authors:** Kathrin Ebner, Franz Schaefer, Max Christoph Liebau, The ARegPKD Consortium, L. A. Eid

**Affiliations:** ^1^Department of Pediatrics, University Hospital of Cologne, Cologne, Germany; ^2^Division of Pediatric Nephrology, Centre for Pediatrics and Adolescent Medicine, Heidelberg University Medical Centre, Heidelberg, Germany; ^3^Center for Molecular Medicine, University Hospital of Cologne, Cologne, Germany; ^4^Nephrology Research Laboratory, Department II of Internal Medicine, University Hospital of Cologne, Cologne, Germany

**Keywords:** ARPKD, ciliopathy, *PKHD1*, fibrocystin, polycystic kidney disease, congenital hepatic fibrosis

## Abstract

Autosomal recessive polycystic kidney disease (ARPKD) is a rare monogenic disease with a severe phenotype often presenting prenatally or in early childhood. With its obligate renal and hepatic involvement, ARPKD is one of the most important indications for liver and/or kidney transplantation in childhood. Marked phenotypic variability is observed, the genetic basis of which is largely unknown. Treatment is symptomatic and largely empiric as evidence-based guidelines are lacking. Therapeutic initiatives for ARPKD face the problem of highly variable cohorts and lack of clinical or biochemical risk markers without clear-cut clinical end points. ARegPKD is an international, multicenter, retro- and prospective, observational study to deeply phenotype patients with the clinical diagnosis of ARPKD. Initiated in 2013 as a web-based registry (www.aregpkd.org), ARegPKD enrolls patients across large parts of Europe and neighboring countries. By January 2017, more than 400 patients from 17 mostly European countries have been registered in the ARPKD registry study with significant follow-up data. Due to comprehensive retro- and prospective data collection and associated biobanking, ARegPKD will generate a unique ARPKD cohort with detailed longitudinal clinical characterization providing a basis for future clinical trials as well as translational research. Hence, ARegPKD is hoped to contribute to the pathophysiological understanding of the disease and to the improvement of clinical management.

## Background

Autosomal recessive polycystic kidney disease (ARPKD) is a severe form of polycystic kidney disease with early manifestation and substantial morbidity and mortality. ARPKD constitutes one of the most important causes of renal replacement therapy and/or liver transplantation in childhood. Hence, the disease has substantial socioeconomic impact despite its low incidence (1 in 20,000 live births) (1).

Autosomal recessive polycystic kidney disease is caused by mutations in a single gene, *polycystic kidney and hepatic disease 1* (*PKHD1*), encoding the ciliary protein fibrocystin/polyductin (2). Fibrocystin is a very large protein with a single transmembrane domain and a short cytoplasmatic part (2). Fitting to the ciliary hypothesis (3) and the obligate renal and hepatic phenotype, fibrocystin is expressed in primary cilia of renal and bile duct epithelial cells (4, 5). Fibrocystin seems to exert some effects via the same intracellular signaling pathways which are also dysregulated in the closely related autosomal dominant polycystic kidney disease (ADPKD) (6, 7), but the specific mechanisms leading to cysts and the peculiar clinical phenotype of the disease are still to be identified and described in detail.

The phenotypic spectrum of ARPKD is broad, even within affected families (8, 9). The disease is associated with bilateral ubiquitous fusiform dilatations of the collecting duct, leading to multiple very small cysts and a massive increase of kidney volume. Unlike in ADPKD, no clear correlation between kidney volume and the decline of kidney function over time has been observed (10–12). Despite massive growth of kidneys that can occur even before birth, renal function is frequently preserved over several months or even years. In patients with prenatally impaired renal function, oligohydramnios may lead to pulmonary hypoplasia with associated pulmonary hypertension. These patients suffer from significant morbidity and mortality. Other patients show late disease onset with a predominantly hepatic phenotype and preserved renal function until adolescent or even adult age (9, 13).

The genotype–phenotype correlation is currently refined to the observation that patients with two truncating mutations in *PKHD1* show a severe phenotype with common perinatal demise, whereas children surviving the postnatal period carry at least one missense mutation (9). However, severe courses have also been described with missense mutations. Interpretation is hampered by the big size of the gene with 67 exons and a complex splicing pattern, the absence of a mutational hotspot, and the multiple private mutations, detected only within individual families. More than 700 variations have been described and classified in the *PKHD1* mutation database (www.humgen.rwth-aachen.de/index.php).

Remarkably, even up to 20% of siblings show marked variance in phenotype (9), indicating that the genotype alone is not sufficient to explain the phenotypic variability in ARPKD (9, 14). The genetic basis for the large inter- and intrafamiliar phenotypic variability is poorly understood. Epigenetic and environmental factors as well as variations in other ciliopathy or PKD genes have been suggested (9, 10, 15). However, no genetic, clinical, or biochemical risk markers predicting disease progression or severity have been identified to date.

The treatment of renal insufficiency, arterial and pulmonary hypertension as well as the management of the hepatic phenotype with congenital hepatic fibrosis, portal hypertension, and cholangitis are largely symptomatic and opinion based, relying on expert recommendations (16). Disease-specific causative treatment options are still lacking, despite tremendous progress in our understanding of ciliary pathomechanisms (17–19). Targeted treatment approaches have emerged for ADPKD (20–25), but the ADPKD trial findings cannot readily be extrapolated to ARPKD. Despite the much more severe phenotype of ARPKD with progression to end-stage renal disease of the majority of the patients within the first two decades of life (9) [as compared to 58.1 years (PKD1) and 79.9 years (PKD2) in ADPKD (26)], the large individual phenotypic variability and the 40-fold lower incidence of ARPKD relative to ADPKD have so far precluded the development of clinical trial programs to evaluate the usefulness of compounds interfering with cyst growth.

Several landmark studies, e.g., by Guay-Woodford and Desmond (27), Bergmann et al. (9), and Adeva et al. (13), have described the clinical course of individuals with ARPKD in European and North American cohorts. Despite these excellent previous works, numerous questions remain regarding long-term clinical outcomes, the relative efficacy of current symptomatic treatment approaches, and the most suitable criteria to define risk-based cohort stratification in future interventional trials.

To tackle these questions and challenges, the ARegPKD registry study was recently initiated by the German Society for Pediatric Nephrology (GPN) and the European Study Consortium for Chronic Kidney Disorders Affecting Pediatric Patients Network (ESCAPE-Network). Here, we report about the current state of progress of this study.

## Design

ARegPKD is an international, multicenter, and observational study that follows both pediatric and adult patients with the clinical diagnosis of ARPKD (28).

Diagnostic criteria are (a) typical findings on renal imaging and (b) one or more of the following criteria:

- Clinical/laboratory signs of hepatic fibrosis
- Hepatic pathology demonstrating ductal plate abnormality
- Absence of renal enlargement and/or multiple cysts in both parents
- Pathoanatomic diagnosis of ARPKD in an affected sibling
- Family history consistent with autosomal recessive inheritance.

Exclusion criteria encompass definite genetic, histological, or clinical proof of other cystic kidney disorders.

Deep phenotypic characterization is ensured by entry of basic data and both retro- and prospective visits. Basic data encompass age and clinical symptoms at primary manifestation as well as perinatal period, genetic testing, and family history. Date of diagnosis and criteria leading to diagnosis are checked for plausibility with other entries. The visits (initial visit/follow-up visits) can be entered retro- and prospectively thereby also enabling inclusion of deceased pediatric patients in order to counteract the problem of underreporting of severely affected and early deceased patients. Visits are planned to be entered annually but can be documented at flexible time intervals, e.g., using longer intervals for retrospective entry of adult patients but more frequent prospective data entries of incident patients. In this way, detailed longitudinal information is provided over decades of patient follow-up for the following type of information:

- Patient status—baseline evaluation (e.g., body measurements, participation in studies, availability of biosamples)
- Renal status including symptoms, radiological findings, and biopsy results
- Extrarenal status regarding liver (symptoms, radiological findings, biopsy results) and other organs (spleen, cardiovascular system, central nervous system, eyes, lungs, bones, blood)
- Laboratory values
- Medications with start and end date, dose
- Therapy including renal replacement therapy, surgical procedures, and other procedures
- Further developments (symptoms, performed diagnostic procedures, any additional information) with inclusion of user-defined comments.

Pseudonymized data are entered into a password-restricted, web-based database (www.aregpkd.org) by authorized medical personnel. Subject pseudonymization is performed at the corresponding center. The database is stored on a safe server of the Cologne University Computing Facilities and secured by use of SSL connections.

Given the heterogeneity of the patient cohort and the variety of entering individuals, automatic checks for coherence, plausibility, and validity were installed according to a detailed data validation plan. Erroneous entries are recognized by application of predefined plausibility ranges for measurements, laboratory values, and medication doses. Queries are sent to local investigators in order to complete data and solve plausibility problems or discrepancies.

The clinical documentation is complemented by prospective collection and storage of biomaterials in a central biorepository. Coded samples are stored at the Hannover Unified Biobank at Hannover Medical School, one of the most advanced biobanks in Europe with high quality and safety standards. Consent process is complete for a large number of research approaches. For any specific subsequent study, this question will be re-evaluated by the study consortium coordinators and, where necessary, a corresponding ethics committee.

Reference histopathology of biopsy samples is offered at the University Hospital of Cologne by an internationally recognized expert for ARPKD.

The study protocol has been approved by the Ethics Commission of the Faculty of Medicine of Cologne University and the Institutional Review Boards of each participating site.

Statistical analysis is performed by a biostatistician of the Institute of Medical Biometry and Informatics, University of Heidelberg, according to a statistical analysis plan defining the detailed descriptive analysis of the clinical courses, the identification of clinical risk factors, and the evaluation of therapeutic approaches.

Researchers, both those who are currently engaged in the registry and external investigators, are encouraged to approach the study consortium coordinators for collaborations with a specific research question that will be discussed during the regular consortium meetings.

Patients and participating centers can continuously follow the progress of recruitment for the registry on the public webpage. There is furthermore a regular newsletter on ARegPKD and PKD aspects in general that is sent to the specific centers, which can be passed on to patients or can be downloaded from the ARegPKD webpage.

## Current Status

**Figure 1:**
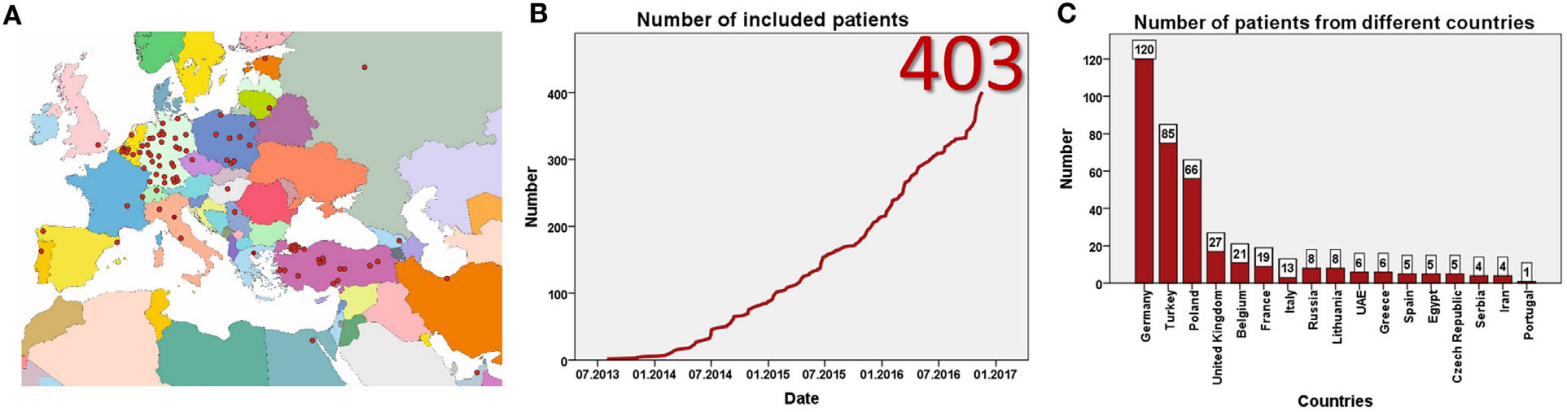
**Registered centers (A) and included number of patients (B)**. Number of included patients from different countries **(C)**.

**Figure 2:**
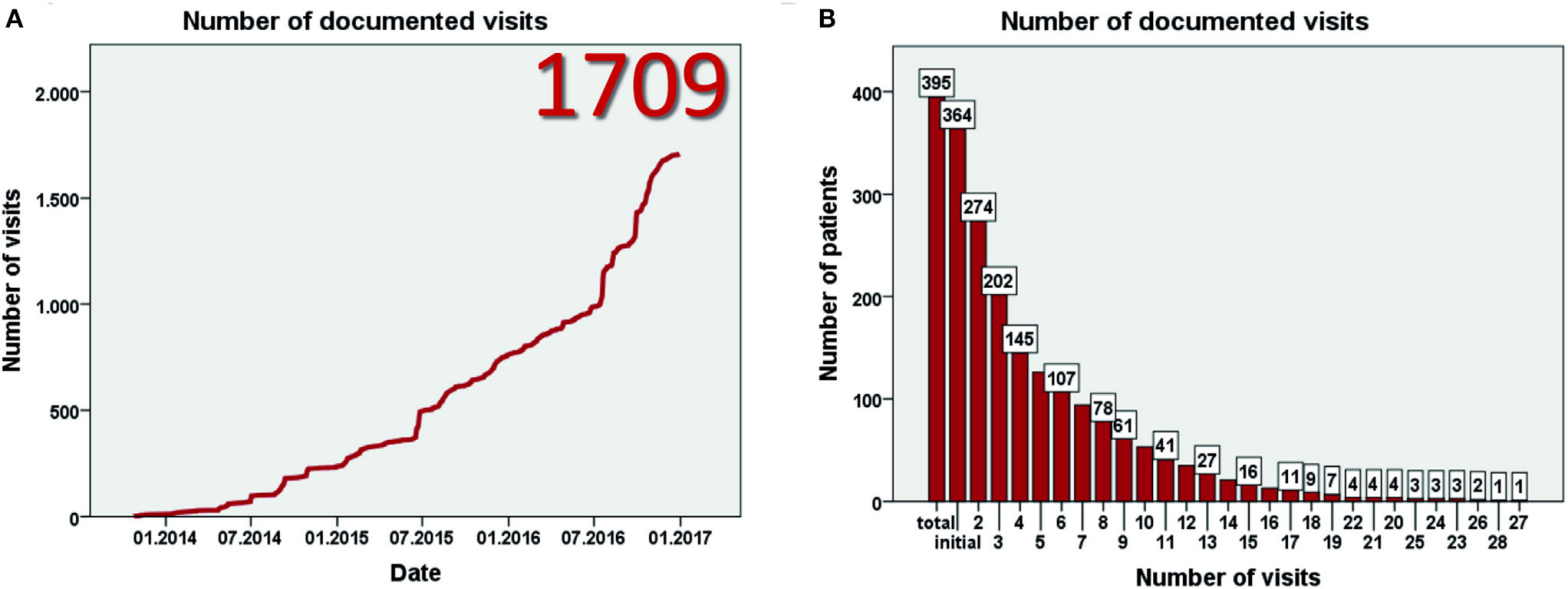
**Number of documented visits (A) and number of visits per patient (B)**.

Data entry started in July 2013. Currently, 93 centers from 23 mostly European countries have registered for the study (Figure 1A). By January 2017, 403 patients have been included (Figure 1B) at 57 centers in 17 countries. Data from eight patients were excluded as patients did not fulfill the inclusion criteria, resulting in data from 395 patients for evaluation. This includes more than 60 adult patients. The largest patient numbers have been enrolled at centers in Germany, Poland, Turkey, and the United Kingdom (Figure 1C). While the majority of patients are currently followed in pediatric nephrology divisions, a substantial number has also been recruited by pediatric gastroenterologists or hepatologists. By January 2017 data from more than 1,700 visits of 364 patients has been collected (Figures 2A,B). To the best of our knowledge, ARegPKD thus represents the largest multinational cohort of ARPKD patients with available detailed clinical data.

Due to enrollment in 17 European and neighboring countries with a cumulative population of more than 300 million people, ARegPKD is gathering a substantial number of ARPKD patients, despite the low incidence of the disease. In addition to answering important questions regarding the natural history of the disease, the international data collection allows a Pan-European reflection on the role of different health-care structures, management approaches, and the large variation in genetic endowment.

Following up patients to adulthood, ARegPKD will provide substantial information regarding the long-term evolution of both early- and late-onset disease. This will allow to address important questions that require observation of disease courses over decades, such as: What are the consequences of early nephrectomy in ARPKD? Does the stringency of blood pressure control influence renal function loss over time? Is the preservation of kidney function differently affected by the type of antihypertensive drugs used? How do extrarenal phenotypes develop? What is the relationship between the renal and the hepatic phenotype in ARPKD? Are there clinical, genetic, or biochemical markers to predict the disease course and to support decision making regarding isolated, combined, and/or sequential liver and kidney transplantation? Are there differences in the management of various disease aspects between regions or countries?

The implementation of comprehensive genotyping will allow the assessment of genotype–phenotype correlations in this clinically deeply characterized cohort. ARegPKD is closely connected to the Network for Early Onset Cystic Kidney Disease (NEOCYST) consortium, in which clinicians, geneticists, and basic scientists have teamed up to study the clinical and genetic overlap of cystic kidney diseases and establish guidelines for diagnostics and standards of care. Within the frame of NEOCYST ARegPKD will also be evaluated by an external advisory board.

The focus and strength of the ARegPKD registry study lies on the stringent collection of detailed longitudinal data in a large patient cohort, which will allow to outline the natural course of the disease and to identify prognostically uniform subcohorts of this heterogeneous disease. Given the observational design of the study, there are obvious weaknesses which include selection bias, incomplete data collection, and operator-dependency of sonographic findings. Yet, the data collected truly represent the “real-life” situation of patients with ARPKD followed in major clinical centers.

In summary, since its start in 2013, ARegPKD has made substantial progress with successful international recruitment of ARPKD patients. Given this progress, ARegPKD will soon provide an observational evidence base to recommendations for the care of patients suffering from ARPKD.

## ARegPKD Consortium

**L. A. Eid**, Dubai, United Arab Emirates; **N. Ranguelov**, Brussels, Belgium; **B. Adams**, Brussels, Belgium; **K. van Hoeck**, Edegem, Belgium; **A. Raes**, Gent, Belgium; **D. Mekahli**, Leuven, Belgium; **L. Collard**, Montegnee, Belgium; **J. Lombet**, Montegnee, Belgium; **J. Maquet**, Montegnee, Belgium; **F. Cachat**, Lausanne, Switzerland; **G. Schalk**, Zurich, Switzerland; **T. Seeman**, Prague, Czech Republic; **N. Ortiz Bruechle**, Aachen, Germany; **K. Zerres**, Aachen, Germany; **J. Thumfart**, Berlin, Germany; **S. Briese**, Berlin, Germany; **U. Querfeld**, Berlin, Germany; **B. Hoppe**, Bonn, Germany; **M. Feldkoetter**, Bonn, Germany; **M. Kirschstein**, Celle, Germany; **G. Gruening**, Celle, Germany; **B. B. Beck**, Cologne, Germany; **T. Benzing**, Cologne, Germany; **R. Buettner**, Cologne, Germany; **J. Dötsch**, Cologne, Germany; **H. Goebel**, Cologne, Germany; **F. Grundmann**, Cologne, Germany; **B. Hero**, Cologne, Germany; **C. Kurschat**, Cologne, Germany; **L. T. Weber**, Cologne, Germany; **B. Mayer**, Dresden, Germany; **J. Weber**, Dresden, Germany; **B. Ritter**, Dresden, Germany; **K. Benz**, Erlangen, Germany; **M. Galiano**, Erlangen, Germany; **A. Tzschoppe**, Erlangen, Germany; **B. Buchholz**, Erlangen, Germany; **R. Buescher**, Essen, Germany; **A. Buescher**, Essen, Germany; **K. Latta**, Frankfurt, Germany; **K. Häffner**, Freiburg, Germany; **M. Pohl**, Freiburg, Germany; **O. Gross**, Goettingen, Germany; **J. Krügel**, Goettingen, Germany; **J. Stock**, Goettingen, Germany; **L. Patzer**, Halle/Saale, Germany; **H. Teichler**, Halle/Saale, Germany; **J. Oh**, Hamburg, Germany; **R. Schild**, Hamburg, Germany; **T. Illig**, Hannover, Germany; **N. Klopp**, Hannover, Germany; **L. Pape**, Hannover, Germany; **S. Wahrendorf**, Hannover, Germany; **W. Bernhardt**, Hannover, Germany; **A. Doyon**, Heidelberg, Germany; **E. Wuehl**, Heidelberg, Germany; **T. Vinke**, Heidelberg, Germany; **A. Sander**, Heidelberg, Germany; **K. Kunzmann**, Heidelberg, Germany; **C. Bergmann**, Ingelheim, Germany; **S. Wygoda**, Leipzig, Germany; **M. Henn**, Leipzig, Germany; **D. Wiemann**, Magdeburg, Germany; **K. Blaschke**, Magdeburg, Germany; **U. Derichs**, Mainz, Germany; **R. Beetz**, Mainz, Germany; **N. Jeck**, Marburg, Germany; **G. Klaus**, Marburg, Germany; **H. Fehrenbach**, Memmingen, Germany; **T. Hampel**, Memmingen, Germany; **S. Zoetler**, Memmingen, Germany; **M. Wallot**, Moers, Germany; **H. Kyrieleis**, Moers, Germany; **B. Lange-Sperandio**, Munich, Germany; **S. Ponsel**, Munich, Germany; **F. Kusser**, Munich, Germany; **J. Hoefele**, Munich, Germany; **B. Uetz**, Munich, Germany; **M. Benz**, Munich, Germany; **S. Schmidt**, Munich, Germany; **C. Huppertz-Kessler**, Munich, Germany; **B. Kranz**, Muenster, Germany; **J. Koenig**, Muenster, Germany; **A. Titieni**, Muenster, Germany; **M. Boeswald**, Muenster, Germany; **H. Staude**, Rostock, Germany; **U. Jacoby**, Rostock, Germany; **D. Wurm**, Saarbrücken, Germany; **H. E. Leichter**, Stuttgart, Germany; **M. Bald**, Stuttgart, Germany; **H. Billing**, Tuebingen, Germany; **M. Gessner**, Tuebingen, Germany; **O. Beringer**, Ulm, Germany; **M.-L. Ilmoja**, Tallinn, Estonia; **N. A. Soliman**, Cairo, Egypt; **M. M. Nabhan**, Cairo, Egypt; **G. Ariceta**, Barcelona, Spain; **L. E. Lara**, Barcelona, Spain; **M. A. Garcia-Gonzalez**, Santiago de Compostela, Spain; **C. Diaz-Rodriguez**, Santiago de Compostela, Spain; **M. Garcia-Vidal**, Santiago de Compostela, Spain; **B. Ranchin**, Lyon, France; **R. Shroff**, London, UK; **R. Sterenborg**, London, UK; **T. Davitala**, Tbilisi, Georgia; **F. Papachristou**, Thessaloniki, Greece; **S. Stabouli**, Thessaloniki, Greece; **P. Sallay**, Budapest, Hungary; **N. Hooman**, Tehran, Iran; **G. Ardissino**, Milano, Italy; **S. Testa**, Milano, Italy; **L. Massella**, Rome, Italy; **F. Emma**, Rome, Italy; **A. Jankauskiene**, Vilnius, Lithuania; **R. Cerkauskiene**, Vilnius, Lithuania; **K. Azukaitis**, Vilnius, Lithuania; **A. Bokenkamp**, Amsterdam, Netherlands; **J. van Wijk**, Amsterdam, Netherlands; **K. Taranta-Janusz**, Bialystok, Poland; **A. Wasilewska**, Bialystok, Poland; **I. Zagozdzon**, Gdansk, Poland; **I. Balasz-Chmielewska**, Gdansk, Poland; **M. Miklaszewska**, Krakow, Poland; **K. Zachwieja**, Krakow, Poland; **D. Drozdz**, Krakow, Poland; **M. Tkaczyk**, Lodz, Poland; **M. Stanczyk**, Lodz, Poland; **P. Sikora**, Lublin, Poland; **M. Zaniew**, Poznan, Poland; **M. Litwin**, Warsaw, Poland; **A. Niemirska**, Warsaw, Poland; **D. Wicher**, Warsaw, Poland; **I. Jankowska**, Warsaw, Poland; **J. Antoniewicz**, Warsaw, Poland; **J. Lesiak**, Warsaw, Poland; **P. Lipinski**, Warsaw, Poland; **M. Szczepanska**, Zabrze, Poland; **P. Adamczyk**, Zabrze, Poland; **A. Morawiec-Knysak**, Zabrze, Poland; **A. Caldas Afonso**, Porto, Portugal; **A. Teixeira**, Porto, Portugal; **G. Milosevski-Lomic**, Belgrade, Serbia; **D. Paripović**, Belgrade, Serbia; **A. Peco-Antic**, Belgrade, Serbia; **L. Prikhodina**, Moscow, Russia; **S. Papizh**, Moscow, Russia; **A. K. Bayazit**, Adana, Turkey; **A. Anarat**, Adana, Turkey; **E. Melek**, Adana, Turkey; **U. S. Bayrakci**, Altindag-Ankara, Turkey; **A. Kantar**, Altindag-Ankara, Turkey; **S. Cayci**, Altindag-Ankara, Turkey; **U. E. Baskin**, Ankara, Turkey; **A. Duzova**, Ankara, Turkey; **A. Yuzbasioglu**, Ankara, Turkey; **A. Soylu**, Balcova, Izmir, Turkey; **S. Kavukcu**, Balcova, Izmir, Turkey; **S. Kalman**, Bestepe-Ankara, Turkey; **H. Evrengül**, Denizli, Turkey; **S. Yüksel**, Denizli, Turkey; **A. Kara**, Firat, Turkey; **M. K. Gurgoze**, Firat, Turkey; **C. Candan**, Istanbul, Turkey; **L. Sever**, Istanbul, Turkey; **S. Caliskan**, Istanbul, Turkey; **N. Canpolat**, Istanbul, Turkey; **S. Emre**, Istanbul, Turkey; **A. Yilmaz**, Istanbul, Turkey; **I. Gökce**, Istanbul, Turkey; **H. Alpay**, Istanbul, Turkey; **N. Akinci**, Istanbul, Turkey; **S. Mir**, Izmir, Turkey; **B. Sozeri**, Izmir, Turkey; **I. Dursun**, Kayseri, Turkey; **H. M. Poyrazoglu**, Kayseri, Turkey; **R. Dusunsel**, Kayseri, Turkey; **H. Nalcacioglu**, Kayseri, Turkey; **Z. Ekinci**, Kocaeli, Turkey; **Y. Tabel**, Malatya, Turkey; **A. Delibas**, Mersin, Turkey; **D. Övünc Hacihamdioglu**, Üsküdar/Istanbul, Turkey; **L. Guay-Woodford**, Washington, DC, USA; **ESCAPE Study Group**; **GPN Study Group**.

## Ethics Statement

Main votum: Ethics committee of the Medical Faculty of the University of Cologne. Written informed consent was obtained prior to participation. Informed consent files were reviewed by Ethics committee. Consent by pediatric patients and parents. Age-adjusted consent forms.

## Author Contributions

KE, FS, and ML drafted the manuscript. FS and ML designed the study. KE, FS, and ML contributed to coordinated European establishment of the study. All the authors reviewed and approved the final manuscript.

## Conflict of Interest Statement

The authors declare that the research was conducted in the absence of any commercial or financial relationships that could be construed as a potential conflict of interest. The handling Editor declared a past co-authorship with one of the authors ML and states that the process nevertheless met the standards of a fair and objective review.
